# The role of the JAK/STAT pathway in the pathogenesis and treatment of post-COVID autoimmune syndromes

**DOI:** 10.3389/fphar.2026.1939700

**Published:** 2026-10-26

**Authors:** Lorenza Bruno, Marta Di Berardino, Giacomo Cafaro, Roberto Dal Pozzolo, Fulvia Ceccarelli, Yehuda Shoenfeld, Roberto Gerli, Elena Bartoloni, Carlo Perricone

**Affiliations:** 1 Rheumatology, Department of Medicine and Surgery, University of Perugia, Piazzale Giorgio Menghini, Perugia, Italy; 2 Division of Rheumatology, Department of Clinical Internal, Anaesthesiologic and Cardiovascular Sciences, Sapienza University, Rome, Italy; 3 Reichman University, Herzliya, Israel; 4 The Zabludowicz Center for Autoimmune Diseases and Rheumatology Unit, Sheba Medical Center, Tel Hashomer, Israel

**Keywords:** autoimmune diseases, cytokine storm, dysautonomia, fibromyalgia, JAK/STAT, Janus kinase inhibitors, post-COVID

## Abstract

The coronavirus disease 2019 (COVID-19) pandemic has not only resulted in acute respiratory illness but has also led to a growing population of individuals affected by persistent, debilitating symptoms, collectively known as post-COVID syndrome or “Long COVID”. This condition presents with a variety of manifestations, including fatigue, joint pain, cognitive disturbances, and dysautonomia, often mimicking autoimmune and neuroinflammatory disorders. Recent research highlights the Janus kinase/signal transducer and activator of transcription (JAK/STAT) signalling pathway as a central mediator in the inflammatory cascade triggered by SARS-CoV-2 infection. This review explores the dual role of the JAK/STAT pathway in both the acute phase of COVID-19 and its potential contribution to post-infectious autoimmune phenomena. We summarize the current understanding of JAK inhibitors (JAKi) as therapeutic agents for mitigating hyperinflammation during active infection and examine their emerging role in the management of post-COVID syndromes, particularly in cases with pulmonary involvement and features overlapping with fibromyalgia and chronic fatigue syndrome. Moreover, we discuss the safety profile of JAKi, their impact on vaccine immunogenicity, and the implications of autoantibody formation targeting the autonomic nervous system. Understanding the immunopathological mechanisms underpinning post-COVID may not only inform therapeutic strategies but also illuminate broader pathways involved in chronic autoimmune conditions.

## 1 Introduction

The novel coronavirus SARS-CoV-2, spread worldwide since late 2019, has caused significant global consequences, not only due to acute infections but also to its long-term health sequelae (López-Sampalo et al., 2022). While the initial focus of the scientific and clinical community was on managing the acute respiratory illness caused by COVID-19, there is now growing recognition and interest in persistent post-infection symptoms, collectively referred to as post-COVID syndrome or “Long COVID” (Raveendran et al., 2021). As defined by the World Health Organization (WHO) in 2021, this syndrome affects individuals with a prior SARS-CoV-2 infection, typically manifesting 3 months after onset and persisting for at least 2 months without an alternative diagnosis (Soriano et al., 2022).

The pathophysiology of post-COVID syndrome is not yet fully understood, but recent findings suggest a complex combination of factors, including persistent immune activation, presence of viral remnants, dysregulated cytokine responses, and possibly autoimmunity (Raveendran et al., 2021; Halpert and Shoenfeld, 2020). Central to many of these immune processes are the Janus kinase (JAK)/signal transducer and activator of transcription (STAT) pathway, a critical signaling cascade for cytokine-mediated immune modulation (Rommasi et al., 2022).

This review explores the multifaceted role of the JAK/STAT signaling axis in the pathogenesis and potential treatment of post-COVID autoimmune syndromes. It also examines the therapeutic implications of JAK inhibitors (JAKi), their benefits in modulating hyperinflammation and post-infection autoimmune sequelae, and their emerging relevance in clinical practice. Special focus is placed on the intersection of post-COVID with conditions including fibromyalgia (FM), chronic fatigue syndrome (CFS), and dysautonomia, disorders that are increasingly reported in the wake of SARS-CoV-2 infection.

## 2 What is post-COVID?

In March 2020, the World Health Organization (WHO) declared a pandemic emergency due to coronavirus disease 2019 (COVID-19), caused by severe acute respiratory syndrome coronavirus 2 (SARS-CoV-2), first reported in China in late 2019 (López-Sampalo et al., 2022). While the majority of patients are asymptomatic or present with mild illness, a subset of patients with COVID-19 develop severe complications, including interstitial lung disease and acute respiratory distress syndrome (ARDS), associated with a high mortality rate (Wu et al., 2020).

The clinical and epidemiological characteristics, pathogenesis, and complications of patients with COVID-19 in the acute phase have been extensively described, however, the long-term effects of the disease remain poorly understood. Since the beginning of the COVID-19 pandemic, many individuals have reported the persistence of a wide range of debilitating symptoms lasting more than 4 weeks, regardless of viral status and the initial disease severity, which cannot be explained by an alternative disorder and which is now called post-COVID syndrome (Raveendran et al., 2021; Pierce et al., 2022). In October 2021, the WHO defined the post-COVID syndrome as the “Illness that occurs in people who have a history of probable or confirmed SARS-CoV-2 infection; usually 3 months from the onset of COVID-19, and symptoms persisting for a minimum of 2 months and cannot be explained by an alternative diagnosis” (Soriano et al., 2022).

Thus, the acute phase of COVID-19 may result in the persistence of one or more symptoms, or the emergence of new symptoms. A wide range of physical and psychological symptoms occurs among people with post-COVID condition. The most frequently reported are fatigue, headache, attention disorder, hair loss, sleep problems, orthostatic intolerance, post-exertional malaise and dyspnea, which can substantially affect everyday functioning. There have also been numerous reports of chronic pain associated with SARS-CoV-2 infection (Nalbandian et al., 2021; Lavin et al., 2023; Chen et al., 2022). Musculoskeletal (MSK) pain-related conditions are among the leading causes of disability globally (Blyth et al., 2019). Furthermore, the substantial healthcare costs for MSK pain and the resulting loss of productivity represent a huge economic burden for societies (Gaskin and Richard, 2012). Other manifestations of post-COVID syndrome may be related to organ-specific injury resulting from viral damage and inflammation, including pulmonary fibrosis, cardiomyopathy, and thromboembolic events (Lee C. C. E. et al., 2021).

The pathogenesis of post-COVID syndrome is complex and remains incompletely understood, with several potentially overlapping mechanisms currently under investigation. Proposed mechanisms include persistence of SARS-CoV-2 or viral antigens in tissue reservoirs, chronic activation and dysregulation of innate and adaptive immune responses, autoimmunity and autoantibody production, endothelial dysfunction and microvascular abnormalities, reactivation of latent viral infections, alterations of the gut microbiome, mitochondrial and metabolic dysfunction, and neuroinflammatory and autonomic nervous system abnormalities (Rommasi et al., 2022; Lai et al., 2023). These mechanisms are not mutually exclusive and may contribute differently across patients, potentially accounting for the considerable clinical heterogeneity of post-COVID syndrome (Nalbandian et al., 2021; Lavin et al., 2023; Chen et al., 2022; Blyth et al., 2019; Gaskin and Richard, 2012; Lee C. C. E. et al., 2021).

Among the immune pathways implicated in post-COVID syndrome, persistent cytokine and interferon dysregulation has received increasing attention. Altered signaling involving type I and type II interferons, IL-1β, IL-6, transforming growth factor-β, nuclear factor-κB, and the JAK/STAT pathway has been described (Rommasi et al., 2022; Lai et al., 2023). Within this broader and multifactorial pathogenic framework, the present review specifically focuses on the potential contribution of JAK/STAT signaling to persistent immune dysregulation and post-COVID manifestations, as well as on the potential therapeutic implications of targeting this pathway. The main clinical and immunopathological features of post-COVID syndrome are summarized in Tables 1, 2.

**TABLE 1:** Overview of clinical features of post-COVID.

Topic	Key concepts	Clinical features
Definition of post-COVID	Persistent symptoms after SARS-CoV-2 infection (Raveendran et al., 2021; Pierce et al., 2022)	Symptoms persist >4 weeks or >3 months; prevalence estimated 2%–15%; severe fatigue, cognitive dysfunction, dysautonomia, dyspnea common (Raveendran et al., 2021; Pierce et al., 2022) Major emerging chronic multisystem syndrome
Most common symptoms	Fatigue, PEM, brain fog (Nalbandian et al., 2021; Lavin et al., 2023; Chen et al., 2022)	Fatigue with post-exertional malaise is dominant; cognitive impairment frequent (Nalbandian et al., 2021; Lavin et al., 2023; Chen et al., 2022) Overlaps with FM, ME/CFS, dysautonomia
Post-COVID and autoimmunity	Autoimmune risk after infection	Increased relative risk of autoimmune diseases; possible triggering/exacerbation of CTDs
Post-COVID in IMIDs	Rheumatic diseases and susceptibility	Patients with inflammatory rheumatic diseases may have increased risk of persistent symptoms Underlying disease activity may confound diagnosis
Musculoskeletal manifestations	Arthralgia, myalgia, arthritis (Nalbandian et al., 2021; Lavin et al., 2023; Chen et al., 2022; Blyth et al., 2019)	Arthralgia common; acute arthritis and myalgia frequently reported (Nalbandian et al., 2021; Lavin et al., 2023; Chen et al., 2022; Blyth et al., 2019)
FM-like syndrome	Central sensitization	Up to 30% may fulfill FM criteria after COVID (Bair and Krebs, 2020; Goldenberg, 2024; Ursini et al., 2021)
Neurocognitive dysfunction	Brain fog and cognitive decline	Persistent deficits in attention, memory, executive function; ICU patients more severely affected Objective measurable cognitive impairment
Sleep disturbance	Insomnia and sleep dysfunction	Poor sleep quality, daytime sleepiness, apnea risk common Sleep disturbance may amplify pain and fatigue
Pulmonary involvement	ILD-like manifestations	Ground-glass opacities and persistent dyspnea may require HRCT follow-up (Lee et al., 2021a) Relevant differential diagnosis in CTD-ILD
Dermatologic manifestations	Chilblains, urticaria, purpura	Wide spectrum of cutaneous findings resembling autoimmune diseases
Management	Multidisciplinary approach	Rehabilitation, pacing, symptom-oriented therapy, rheumatologic evaluation when needed No universally effective treatment available
Therapeutic approaches	Anti-inflammatory/immunomodulatory therapies	Colchicine and cytokine-targeting strategies discussed; evidence still limited Future role for targeted immunotherapy uncertain

List of abbreviations: CFS, chronic fatigue syndrome; COVID, coronavirus disease; CTD, connective tissue disease; FM, fibromyalgia; HRCT, high-resolution computed tomography; ICU, intensive care unit; ILD, interstitial lung disease; IMID, immune-mediated inflammatory diseases; ME, myalgic encephalomyelitis; PEM, post-exertional malaise.

**TABLE 2:** Overview of immunological and pathophysiological features of post-COVID.

Topic	Key concepts	Main findings
Pathogenesis	Viral persistence, immune dysregulation, mitochondria	Persistent inflammation, mitochondrial dysfunction, cGAS-STING activation, endothelial injury Multifactorial disease model
Cytokines and inflammation	IL-6, TNF-α, interferons (Rommasi et al., 2022; Lai et al., 2023)	Hyperinflammation and cytokine dysregulation linked to disease severity and persistence Supports rationale for immunomodulatory approaches
Autoantibodies	aPL and immune dysregulation	Increased antiphospholipid antibodies after COVID, though not clearly associated with thrombosis (Dani et al., 2021; Bornstein et al., 2022) Suggests transient immune activation
Immune profiling	Persistent immune activation	Altered cytokines, abnormal Tfh/Treg balance, autoantibodies in Post-COVID Suggests chronic immune dysregulation
Autonomic dysfunction	Anti-GPCR antibodies (Dotan et al., 2022)	Possible autoimmune dysautonomia mechanisms

List of abbreviations: COVID, coronavirus disease; Anti-GPCR, anti–G protein-coupled receptor antibodies; aPL, antiphospholipid antibodies.

## 3 Pathophysiological mechanisms of post-COVID syndrome: from immune dysregulation to dysautonomia

Symptoms occurring in post-COVID syndrome overlap with those of myalgic encephalomyelitis/chronic fatigue syndrome (ME/CFS), including disabling fatigue, autonomic dysfunction, sleep disturbances, and cognitive impairment, often exacerbated by exertion. A significant proportion of post-COVID patients have been found to meet the diagnostic criteria for FM (Bair and Krebs, 2020; Goldenberg, 2024). This is a chronic disorder characterised by widespread musculoskeletal pain, fatigue and a variety of additional symptoms, including sleep disturbances, cognitive complaints, mood alterations, and other somatic manifestations (Bair and Krebs, 2020). There are no specific medical or instrumental tests to diagnose the condition (Galvez-Sánchez and Reyes Del Paso, 2020). Patients with FM experience increased sensitivity to painful stimuli (hyperalgesia) and painful responses elicited by non-painful stimuli (allodynia), suggesting that FM is a disorder of central pain processing rather than a localized disease in the part of the body where the pain is experienced (Clauw, 2009). Several studies linked cytokines with FM syndrome (Wallace et al., 2001). García et al. (2014) demonstrated that patients with FM had elevated serum levels of several inflammatory chemokines, including TARC, MIG, MDC, I-TAC, and eotaxin as well as some of the proinflammatory cytokines involved in COVID-19 manifestations, such as IL-1 and IL-6 (Rommasi et al., 2022), may contribute to the pathogenesis of FM (Theoharides et al., 2019). Ursini et al. (2021) reported the results of a web-based survey aimed at investigating the prevalence of FM developed after symptomatic COVID-19; the secondary aim was to investigate predictive factors for the development of post-COVID-19 FM syndrome. It has been shown that 30.7% of patients who had recovered from COVID-19 fulfilled the American College of Rheumatology (ACR) criteria for FM, 43.4% of which were men. It has been hypothesized that COVID-19 infection may cause an alteration of the autonomic nervous system, mediated by virus or immune mechanisms, resulting in transient or chronic orthostatic intolerance syndromes (orthostatic hypotension, vasovagal syncope and postural orthostatic tachycardia syndrome) (Goldstein, 2020).

Indeed, FM, CFS and post-COVID syndrome are characterized by typical clinical symptoms of dysautonomia (Mahroum and Shoenfeld, 2022; Ohry and Shoenfeld, 2024). It is established that these autonomic disorders are associated with autoantibodies, for example, to α-/β-adrenoceptors and muscarinic receptors (Dani et al., 2021). Bornstein et al. (2022) found similar elevation of these autoantibodies in post-COVID patients and in patients with CFS. In another study (Wallukat et al., 2021), functionally active autoantibodies against the autonomic nervous system were found in post-COVID syndrome patients. In detail, all patients had between two to seven different antibodies that acted as receptor agonists, such as β2-adrenoceptor, α1-adrenoceptor, and Angiotensin II receptor type 1 receptor. Finally, Dotan et al. (2022) identified nearly 20 distinct autoantibodies against G protein-coupled receptors (GPCRs) of the nervous system and renin-angiotensin system-related molecules that were significantly associated with COVID-19 severity and may contribute to autonomic nervous system dysfunction in post-COVID syndrome. The proposed interplay between persistent immune dysregulation, JAK/STAT activation, and autonomic nervous system dysfunction in post-COVID syndrome is summarized in Figure 1. The possible involvement of autoantibodies against the autonomic nervous system provides a rationale for investigating immunomodulatory approaches in selected patients with post-COVID syndrome; however, their clinical efficacy remains to be established.

**FIGURE 1:**
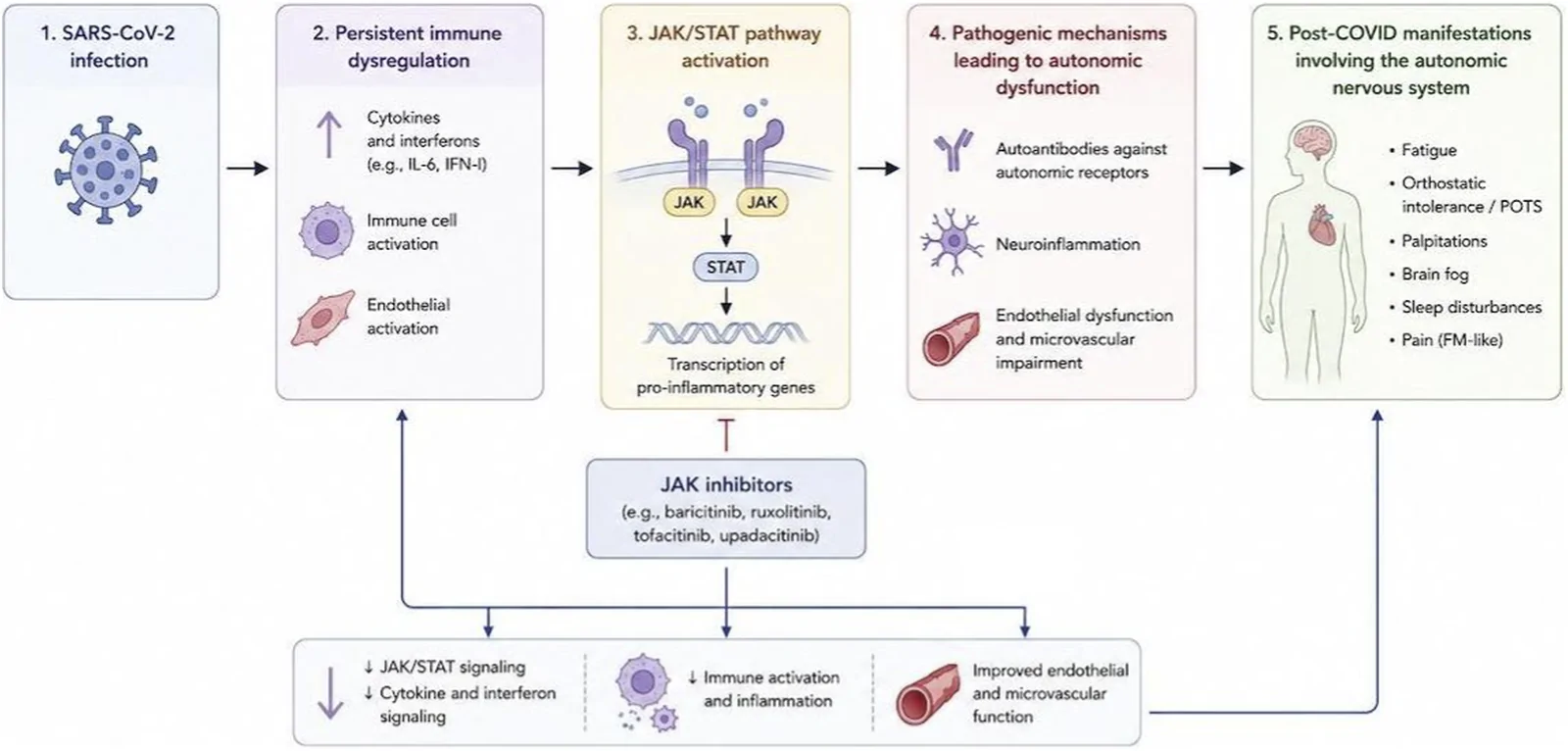
Proposed pathogenesis of post-COVID syndrome involving the autonomic nervous system. SARS-CoV-2 infection may trigger persistent immune dysregulation, characterized by increased cytokine and interferon signaling, immune-cell activation, and endothelial dysfunction, leading to sustained activation of the JAK/STAT pathway. Downstream immune-mediated mechanisms, including autoantibody production, neuroinflammation, and endothelial and microvascular dysfunction, may contribute to autonomic nervous system dysfunction and related post-COVID manifestations. JAK inhibitors may counteract these processes by reducing JAK/STAT signaling and downstream cytokine and interferon-mediated inflammation, potentially improving autonomic and systemic manifestations. The figure was originally created by the authors and subsequently graphically refined using ChatGPT (GPT-5.5, OpenAI). The final version was reviewed and approved by the authors to ensure the accuracy of its scientific content. List of abbreviations: FM = fibromyalgia; POTS = postural orthostatic tachycardia syndrome.

Beyond autoantibodies, several other mechanisms are supposed to contribute to autonomic dysfunction, FM-like symptoms and manifestations of ME/CFS in the post-COVID syndrome. Several different pathogenetic hypotheses have been suggested, including the imbalance between sympathetic and vagal tone, neurotransmitter dysregulation, endothelial and microvascular dysfunction, low-grade inflammation generated by the persistence of viral products or remnants, persistent immune dysregulation, hyperactivation of cGAS–STING signaling, as well as the persistence of elevated levels of cytokines and pro-inflammatory molecules, the activation of interferon signaling, and the exhaustion of both T cells and B cells (Pan et al., 2021; Wirth and Scheibenbogen, 2026; Vallée, 2021; Phetsouphanh et al., 2022; Queiroz et al., 2024; Peluso and Deeks, 2024; Zollner et al., 2022; Schultheiß et al., 2023; Peluso et al., 2024; Skevaki et al., 2025; Berthelot et al., 2020; Aid et al., 2026; Springe et al., 2026; Fogarty et al., 2021). Activation of cytokine and interferon signaling pathways may contribute to these abnormalities, highlighting the potential importance of JAK/STAT-dependent mechanisms.

Dysautonomia is characterized by an imbalance between sympathetic and parasympathetic activity and has been linked to fatigue in several immune-mediated disorders. Emerging evidence suggests that dysautonomic symptoms may underlie neurotransmitter dysregulation, increased sympathetic activation, and reduced vagal tone (Pan et al., 2021). Furthermore, alterations in serotonergic, GABAergic and glutamatergic signaling, already described in ME/CFS, may also contribute to fatigue and dysautonomia in post-COVID patients (Wirth and Scheibenbogen, 2026).

At the same time, SARS-CoV-2 can influence the nervous system through both direct and indirect neuroinvasive mechanisms. The ACE2 and neuropilin-1 (NRP-1) receptors, expressed in neurons, glial cells and the olfactory epithelium, appear to promote viral neurotropism and central nervous system involvement. Persistent viral presence in tissues may promote endothelial damage, disruption of the blood-brain barrier and chronic neuroinflammation, potentially explaining some of the persistent neurological symptoms observed in post-COVID syndrome (Vallée, 2021).

Not only neurotransmitter dysfunction but even persistent immune dysregulation has emerged as a distinctive feature of long COVID (Phetsouphanh et al., 2022). Patients with post-COVID syndrome show increased activation of the cGAS–STING pathway and elevated levels of interferon, which may contribute to the persistence of low-grade chronic inflammation (Queiroz et al., 2024). Phetsouphanh et al., 2022 demonstrated that patients with long COVID exhibited persistent activation of innate immune cells, depletion of naïve T and B lymphocytes, and sustained elevation of type I (IFN-β) and type III interferons, which remained markedly increased up to 8 months after the acute infection.

Moreover, recent evidence has shown that viral reservoirs or viral remnants remain in tissues, such as the gastrointestinal tract, lymphoid organs, the nervous system, and plasma (Peluso and Deeks, 2024; Zollner et al., 2022; Schultheiß et al., 2023; Peluso et al., 2024; Skevaki et al., 2025). Continuous exposure to viral antigens may therefore promote the activation of the innate immune system via pathways that include cGAS–STING signaling (Berthelot et al., 2020), leading to release of interferons and production of pro-inflammatory cytokines, contributing to neuroinflammation and chronic pain.

Long COVID has also been associated with elevated levels of type I and III interferons, IL-6 and pentraxin-3, alongside persistent activation and exhaustion of CD8^+^ T cells, plasmacytoid dendritic cells and monocytes (Skevaki et al., 2025). Aid et al. (2026) reported increased IL-6 and JAK/STAT pathway activation in patients with post-COVID syndrome. The authors also observed that persistent IFNγ pathway activation was consistently linked to reduced T cell activity and features of T cell exhaustion, suggesting that prolonged immune stimulation may contribute to T cell dysfunction; comparable observations have been reported by Springe et al. (2026), concerning both T cells and B cells.

Endothelial dysfunction and microvascular abnormalities have also been increasingly implicated in post-COVID syndrome (Fogarty et al., 2021). SARS-CoV-2 induces endothelial damage that may compromise tissue perfusion and oxygen delivery, contributing to the fatigue, cognitive impairment and exercise intolerance commonly observed in dysautonomia and ME/CFS-like conditions (Yanai et al., 2024). Furthermore, chronic endothelial activation may further amplify inflammatory and thrombo-inflammatory responses.

Overall, these mechanisms support the hypothesis that post-COVID syndrome is a chronic, multifactorial neuroimmune condition characterized by persistent activation of the innate immune system, dysregulation of interferon signaling, neuroinflammation, endothelial dysfunction and abnormalities in the adaptive immune system (Figure 2). These mechanisms likely interact with autonomic nervous system dysfunction and may contribute to chronic pain syndromes such as FM and ME/CFS. It is worth noting that many of these inflammatory mediators, particularly type I and III interferons and IL-6, converge on the JAK/STAT signaling pathway, supporting its possible central role in the pathogenesis of post-COVID syndrome and highlighting it as a possible therapeutic target.

**FIGURE 2:**
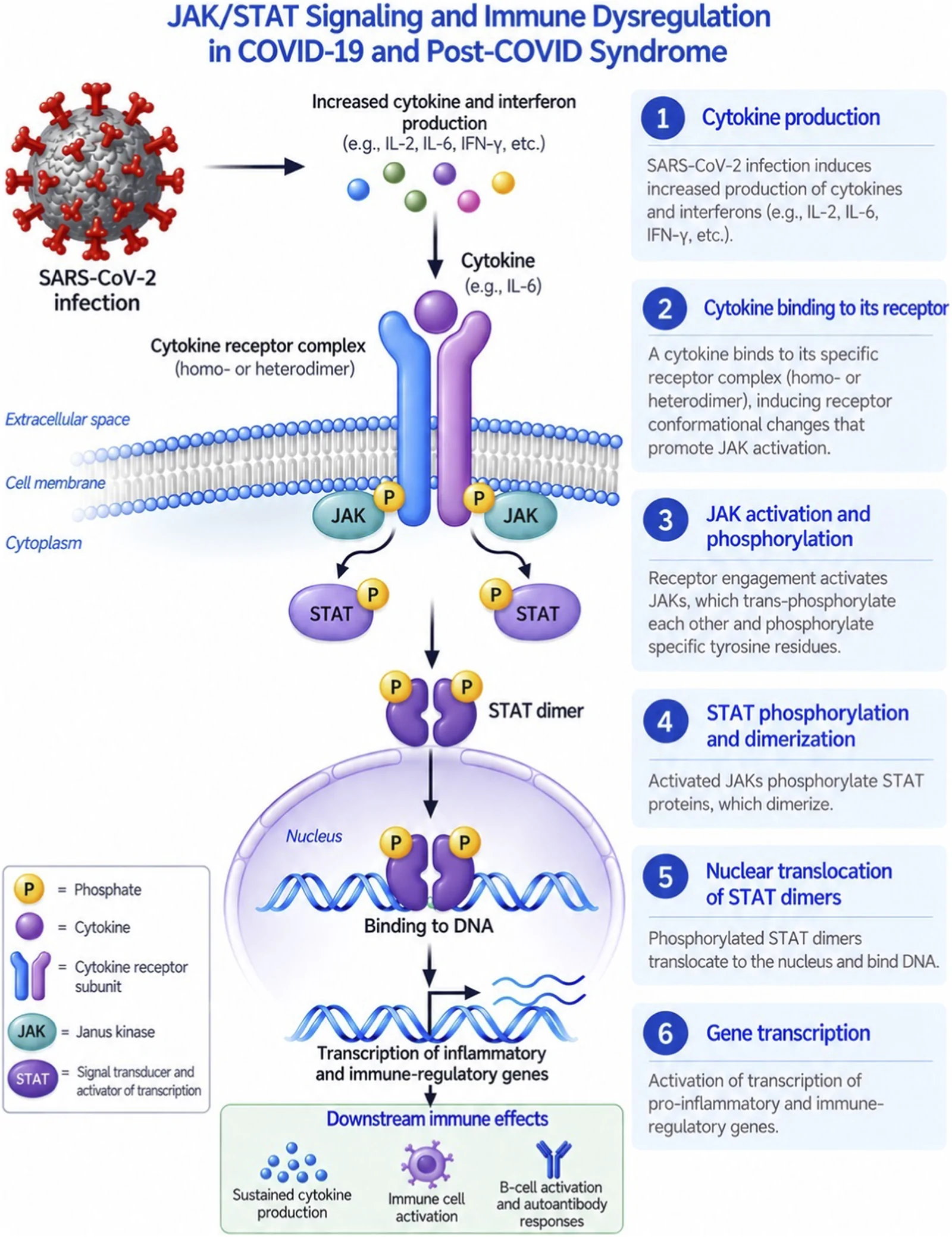
JAK/STAT signaling and immune dysregulation in COVID-19 and post-COVID syndrome. SARS-CoV-2 infection promotes the release of several cytokines, including IL-2, IL-4, IL-6, IL-10, and type I and II interferons, which bind to their respective receptors and activate receptor-associated Janus kinases (JAKs). Activated JAKs phosphorylate STAT proteins, leading to their dimerization and subsequent translocation into the nucleus. STAT dimers bind to DNA and regulate the transcription of inflammatory and immune-regulatory genes, thereby contributing to cytokine production and immune-cell activation. Persistent dysregulation of these pathways may also indirectly promote B cell activation and autoantibody responses, potentially contributing to the autoimmune and inflammatory manifestations of post-COVID syndrome. The figure was originally created by the authors and subsequently graphically refined using ChatGPT (GPT-5.5, OpenAI). The final version was reviewed and approved by the authors to ensure the accuracy of its scientific content.

## 4 JAK inhibitors in COVID-19

SARS-CoV-2 infection induces hyperinflammation with massive inflammatory cytokine production, including IL-1ɑ/β, IL-2, IL-6, IL-17 and TNF-ɑ, leading to an uncontrolled systemic inflammatory reaction known as cytokine storm. The cytokine storm is implicated in the immunopathogenesis of various disease complications, like ARDS and multiorgan failure, even resulting in death (Rommasi et al., 2022; Mehta et al., 2020). The JAK/STAT pathway is implicated in the SARS-CoV-2-induced cytokine storm (Seif et al., 2020; Ravid et al., 2022; Gotthardt et al., 2019; Matsuyama et al., 2020). Persistent cytokine-mediated immune activation may also contribute to autonomic dysfunction, thereby providing a rationale for therapeutic modulation of the JAK/STAT pathway. Furthermore, the JAK/STAT pathway may be implicated in virus entry into target cells. Indeed, the role of JAK signaling in the transcription and activation of ACE2 has been reported in some studies (Luo et al., 2021; Lee H. K. et al., 2021). Consequently, JAK inhibition has emerged as an effective therapeutic strategy in selected patients with severe COVID-19 (Horwitz et al., 2022). JAKs are a family of non-receptor protein tyrosine kinases that mediate the signaling of numerous cytokines implicated in the pathogenesis of autoimmune diseases (Xu et al., 2020). JAKs are constitutively associated with specific cytokine receptors and are activated following binding of the corresponding cytokine or growth factor to its receptor (Yamaoka et al., 2004). Following receptor engagement, activated JAK kinases phosphorylate tyrosine residues on STAT proteins, determining dimerization of the STATs and their translocation into the nucleus, allowing for transcription of their target genes, mainly transcription of proinflammatory genes (Morris et al., 2018). The JAK family has four members: JAK1, JAK2, JAK3 and tyrosine kinase 2 (TYK2). JAK1, JAK2 and TYK2 are fairly ubiquitously expressed in mammalian cells, while JAK3 is predominantly expressed in hematopoietic cells (Alicea-Velázquez and Boggon, 2011). Targeting the JAK/STAT pathway has led to the development of small molecule kinase inhibitors. JAK inhibitors are orally administered, characterized by rapid absorption and relatively short half-lives, allowing rapid onset and reversibility of their pharmacological effects. Despite sharing a common mechanism of action, individual JAK inhibitors differ in their pharmacokinetic profiles, including hepatic metabolism, renal elimination, and susceptibility to drug–drug interactions (Antonioli et al., 2024). From a pharmacodynamic perspective, their activity is primarily determined by relative selectivity for the four JAK family members (JAK1, JAK2, JAK3, and TYK2). First-generation JAK inhibitors generally target multiple JAK isoforms, whereas newer agents have been developed with greater selectivity for individual family members, particularly JAK1 or TYK2 (Yamaoka and Oku, 2023; Virtanen et al., 2024). However, selectivity is relative rather than absolute and depends on drug concentration and cellular context (Virtanen et al., 2024; Traves et al., 2021). These pharmacodynamic differences result in variable modulation of cytokine signaling, including pathways mediated by interleukins and interferons, and may influence both therapeutic effects and safety profiles. Notably, tofacitinib inhibits predominantly JAK1/JAK3 with activity against JAK2, baricitinib preferentially targets JAK1/JAK2, whereas upadacitinib was developed as a JAK1-selective inhibitor (Traves et al., 2021). These differences result in distinct patterns of cytokine inhibition, since individual cytokine receptors signal through different combinations of JAK proteins (Antonioli et al., 2024; Virtanen et al., 2024). At clinically relevant concentrations, these agents modulate signaling pathways involving IL-6 and interferons (Traves et al., 2021), which are of particular interest in the context of the persistent immune dysregulation described in post-COVID syndrome.

Food and Drug Administration (FDA) approval of baricitinib, a JAK1/2 inhibitor, to treat hospitalized COVID-19 adult patients requiring supplemental oxygen, non-invasive or invasive mechanical ventilation, or ECMO, was supported by two phase 3 double-blind, randomized, placebo-controlled trials: ACTT-2 and COV-BARRIER. In the ACTT-2 trial (Kalil et al., 2021), baricitinib plus remdesivir was superior to remdesivir alone in reducing recovery time and accelerating improvement in clinical status among patients with COVID-19, especially in the subgroup that required high-flow oxygen or noninvasive ventilation. In the COV-BARRIER (Marconi et al., 2021), treatment with baricitinib in addition to standard of care (including dexamethasone) had a similar safety profile to that of standard of care alone and was associated with reduced mortality in hospitalized adults with COVID-19. Subsequent evidence has further supported the efficacy and safety of baricitinib in acute COVID-19. A recent meta-analysis (Sun et al., 2023) was performed to investigate the effects of baricitinib for the treatment of hospitalized adults with COVID-19: baricitinib did not increase the incidence of adverse reactions, but it reduced the mortality of COVID-19 patients, not including the critically ill. Similarly, in another meta-analysis, Song et al. (2023) further confirmed baricitinib improving of clinical outcomes in patients with severe COVID-19. The analysis included six studies, involving overall 920 patients, and showed that baricitinib significantly reduced both mortality and the need for mechanical ventilation compared with standard therapy, while maintaining a comparable safety profile and without significantly affecting hospital stay duration (Song et al., 2023). The therapeutic benefit of baricitinib is mainly attributed to its inhibition of the JAK/STAT signaling pathway, resulting in attenuation of the cytokine-mediated hyperinflammatory response associated with severe COVID-19 (Bragazzi et al., 2022; Zandman-Goddard et al., 2022).

Additional data on the immunological effects and safety of baricitinib in acute COVID-19 have emerged from a sub-study of the Bari-SolidAct trial, which evaluated vaccinated patients with severe or critical disease. Despite previous concerns regarding a possible increase in serious adverse events (SAEs) among vaccinated individuals receiving baricitinib, the study identified no clear mechanistic association between vaccination status and the safety profile of the drug. Baricitinib reduced markers of T cell and monocyte activation without impairing antibody responses or viral clearance. However, the limited sample size warrants caution in interpreting these findings (Viermyr et al., 2025).

Overall, these findings support baricitinib as an effective and safe adjunctive treatment for hospitalized patients with severe SARS-CoV-2 infection. Consistent with this evidence, the European League Against Rheumatism (EULAR) (Alunno et al., 2022), in 2021 update on the use of immunomodulatory agents as treatment of COVID-19, has suggested the use of baricitinib in combination with glucocorticoids in moderate to severe and critical COVID-19.

The established efficacy of JAK inhibition in acute COVID-19, together with emerging evidence of persistent JAK/STAT pathway activation in post-COVID syndrome, provides a rationale for exploring JAK inhibitors as a potential therapeutic strategy in this setting. Singh et al. (2023) conducted the first study to evaluate the role of JAKi in the management of post-COVID illness. In this retrospective single-center case-controlled study, they recruited patients belonging to the category of persistence of COVID-19 symptoms beyond 4 weeks of illness with pulmonary involvement but only those patients who had turned negative for SARS-CoV-2 by reverse transcription-polymerase chain reaction. All patients required oxygen or other respiratory support during the active COVID-19 phase. All eligible cases had received corticosteroid at a dose of 0.5–0.75 mg per kg of body weight, a few cases were also managed with baricitinib or tofacitinib. Corticosteroids were rapidly tapered off in patients who were started on JAKi for 7–10 days. In patients who did not receive JAKi, corticosteroids were continued for 3–4 weeks. The primary outcome was to compare the proportion of patients who were able to maintain oxygen saturation ≥95% with any oxygen supplementation in the two groups. After 3–4 weeks, nearly all patients were off oxygen support in both groups. In terms of efficacy outcomes, it is worth noting that JAKi were associated with a higher proportion of patients being successfully weaned from oxygen therapy, although the difference was not statistically significant. Furthermore, JAKi were associated with a significantly lower number of adverse events compared to corticosteroids.

More recently, Jyonouchi et al. (2025) reported three patients with long COVID and predominant neuropsychiatric manifestations who were treated with upadacitinib, a selective JAK1 inhibitor. Two patients had a pre-existing diagnosis of autism spectrum disorder, while the third was a typically developing patient. Long COVID was considered to have triggered or exacerbated encephalopathy-like manifestations, with evidence of persistent innate immune activation involving type I IFN signaling. Treatment with upadacitinib was associated with improvement in neuropsychiatric and behavioral symptoms and, in the patients in whom immune profiling was performed, with a reduction in monocyte inflammatory cytokine responses. These findings provide a mechanistic rationale for JAK1 inhibition in patients with persistent IFN-driven immune activation. However, the evidence is limited to three clinically heterogeneous cases, two with pre-existing autism spectrum disorder, and is further limited by concomitant immunomodulatory treatments and the absence of a control group. Thus, although these observations support further investigation of JAK1 inhibition in selected long COVID phenotypes, prospective controlled studies are needed to confirm its efficacy and safety.

Taken together, these preliminary observations suggest that JAK inhibition may warrant further investigation across different post-COVID phenotypes, potentially including both persistent pulmonary manifestations (Singh et al., 2023) and neuroinflammatory presentations (Jyonouchi et al., 2025). However, the currently available evidence remains exploratory and does not yet support routine use of JAK inhibitors in post-COVID syndrome.

Moreover, among the FDA-approved JAK inhibitors, ritlecitinib and deuruxolitinib are relatively recent additions to the therapeutic landscape. Ritlecitinib is an irreversible inhibitor with preferential activity against JAK3 and is approved for the treatment of severe alopecia areata in adults and adolescents aged 12 years and older (King et al., 2023) (KING). Deuruxolitinib is a selective JAK1/JAK2 inhibitor approved for the treatment of severe alopecia areata in adults (King et al., 2024) (KING2). Their distinct kinase selectivity profiles expand the therapeutic spectrum of JAK inhibition and may result in different pharmacodynamic effects compared with other JAK inhibitors. However, clinical evidence regarding their use in COVID-19 or post-COVID syndrome is currently lacking. Therefore, their potential role in post-COVID manifestations remains speculative and would require dedicated clinical investigation.

## 5 Side effects of the use of JAK inhibitors

Drugs that target the JAK/STAT signaling pathway, including JAKi, have demonstrated clinical utility in managing hyperinflammation during COVID-19. There are possible adverse effects of drugs that affect JAK/STAT signaling during COVID-19. JAKi, like all immunosuppressive drugs, can raise the risk of infections and the risk of serious infections. The most frequent infections associated with JAK inhibitors include pneumonia, nasopharyngitis, urinary tract infections, cellulitis and reactivation of Varicella zoster virus (Calvet et al., 2021). Recently, JAKi have come under scrutiny as a class due to a potential risk of venous thromboembolism (VTE) and cardiovascular (CV) events (Shah et al., 2024). Indeed, concerns have been raised about the possibility that the use of JAKi in rheumatoid arthritis (RA) may further increase those risks. A post-market safety trial (ORAL Surveillance trial) (Ytterberg et al., 2022) of patients >50 years of age with active RA and at least one additional CV risk factor, has confirmed a statistically significant increased risk of VTE and CV events, particularly at higher dosages of tofacitinib. Based on these data, the FDA recommended a black box label to warn prescribers of this risk, that was extended across several types of JAKi. A similar warning from the FDA and the European Medicines Agency (EMA) was added for baricitinib (Paroli et al., 2024). Despite these regulatory concerns, several studies have failed to corroborate a consistent increase in thromboembolic risk across all patient populations, generating ongoing debate within the medical community. Regarding COVID-19, vaccines were developed at an unprecedented pace to help control the pandemic. Although mRNA-based SARS-CoV-2 vaccines provide effective protection in immunocompetent individuals, their immunogenicity in immunosuppressed patients remains less well established. Immunomodulatory treatments may reduce the immunogenicity of mRNA COVID-19 vaccination, inhibiting processes that are necessary for an effective vaccine response due to the numerous cellular and immunological effects (van Sleen et al., 2023). Patients with inflammatory rheumatic diseases on immunomodulatory treatments may be less protected. Raptis et al. (2022) conducted a long-term, model-based comparison of the humoral immune response following two dose vaccination in patients with immune rheumatic diseases on different immunomodulatory drugs. Among monotherapies, impaired antibody responses were observed in patients on rituximab, abatacept, JAKi, and TNF inhibitors. Regarding JAKi, Seror et al. (2022) reported immune responses to COVID-19 vaccination in patients treated with JAK inhibitors using the MAJIK-SFR Registry (a nationwide, multicentre, prospective study including patients who initiated JAKi for RA or psoriatic arthritis). Individuals treated with JAKi showed a significantly lower antibody response to SARS-CoV-2 vaccination and the rate of non-responders was higher with upadacitinib. No other parameters, including concomitant use of methotrexate, corticosteroids, dose of JAKi, disease activity, or type of vaccine were associated with non-response. Similar results were found by Schäfer et al. (2022), but in this case the analysis revealed that combination treatment with both JAKi and MTX significantly impaired the immune response compared with medication with JAKi only. In line with these results are those presented by Iancovici et al. (2022): the authors found that RA patients treated with JAKi showed reduced humoral responses following BNT162b2 vaccination, including lower anti-spike IgG levels and impaired viral neutralization capacity compared with healthy controls. In addition, B cell activation markers, such as IRF4, AID, and BCL6, were reduced, suggesting impaired germinal center responses and antibody production (Iancovici et al., 2022). These findings indicate that JAKi may negatively affect vaccine-induced immunity in RA patients, supporting the need for optimized vaccination strategies and potential booster doses in this population. In contrast, Furer et al. (2021) concluded that the attenuating effects of JAKi on vaccine-induced antibody response were generally modest, suggesting that a majority of patients still achieve protective immunity. Based on the abovementioned data, the ACR guidance recommended withholding JAK inhibitors for 1-2 weeks after each COVID-19 vaccine dose, when disease activity allowed (Curtis et al., 2023).

Therefore, while JAKi remain valuable tools in the treatment of autoimmune diseases and in the management of COVID-19-associated hyperinflammation, their use requires careful consideration of potential risks, including infection, thromboembolic events, and attenuated vaccine responses. As the medical community continues to gather data on long-term safety and efficacy, particularly in the context of emerging infections and vaccination strategies, personalised risk-benefit assessments and updated clinical guidelines will be essential for optimizing outcomes in patients receiving JAKi.

## 6 Conclusion

Post-COVID syndrome represents a complex and heterogeneous condition characterised by persistent immune dysregulation, chronic inflammation, and possible autoimmune mechanisms. The JAK/STAT signaling pathway appears to play a central role in these processes, linking cytokine activation, interferon responses, and tissue damage. The clinical overlap between post-COVID, FM, CFS, and dysautonomia further supports the hypothesis of a shared neuroimmune and autoimmune background.

JAK inhibitors have emerged as promising therapeutic agents due to their ability to modulate multiple inflammatory pathways simultaneously. Although data from the acute phase of COVID-19 supports their efficacy in controlling hyperinflammation, their role in post-COVID manifestations remains preliminary and requires further investigation. Safety concerns, including infection risk, thromboembolic events, cardiovascular complications, and reduced vaccine immunogenicity, must also be carefully evaluated.

Overall, a better understanding of the immunopathogenesis of post-COVID syndrome and the identification of specific inflammatory or autoimmune endotypes will be essential to guide personalised therapeutic strategies and clarify the future role of JAKi in this rapidly evolving field.
